# Propofol and Glioblastoma Biology: A Narrative Review of Perioperative Clinical Evidence and PI3K/AKT‐Wnt‐miRNA, GABA‐A, and Immune‐Checkpoint Pathways

**DOI:** 10.1155/bmri/4808235

**Published:** 2026-09-28

**Authors:** Maryam Imani, Marzieh Lotfi, Seyed Hossein Shahcheraghi

**Affiliations:** ^1^ Reproductive Immunology Research Center, Shahid Sadoughi University of Medical Sciences, Yazd, Iran, ssu.ac.ir; ^2^ Abortion Research Center, Reproductive Sciences Institute, Shahid Sadoughi University of Medical Sciences, Yazd, Iran, ssu.ac.ir; ^3^ Department of Molecular Medicine, School of Advanced Medical Technologies, Shahid Sadoughi University of Medical Sciences, Yazd, Iran, ssu.ac.ir; ^4^ Infectious Diseases Research Center, Shahid Sadoughi Hospital, Shahid Sadoughi University of Medical Sciences, Yazd, Iran, ssu.ac.ir

**Keywords:** anesthetic, glioblastoma multiforme, molecular and clinical effects, propofol

## Abstract

Glioblastoma multiforme (GBM) is the most prevalent and aggressive primary brain tumor, with a median survival of approximately 15 months despite current multimodal therapies. Recent clinical observations have suggested that the intravenous anesthetic propofol may be used to examine whether anesthetic agents can affect glioblastoma behavior. Several retrospective studies reported that, in surgical settings, propofol‐based total intravenous anesthesia (TIVA) is associated with reduced operative time, faster recovery, lower intraoperative blood loss, and decreased postoperative complications such as seizures and cerebral edema, benefits particularly evident in patients with low Karnofsky Performance Scores. However, current clinical evidence remains limited, and the reported effects on survival and recurrence are still inconsistent. Preclinical studies showed that propofol′s antitumor potential is mediated through modulation of the PI3K/AKT–Wnt–miRNA axis, reduction of oxidative stress, regulation of immune checkpoint molecules, and restoration of temozolomide sensitivity in resistant cells. However, some reports indicate that propofol may also induce stem‐like features via GABA‐A receptor activation, underscoring its complex and context‐dependent nature. This narrative review summarizes the current molecular and clinical evidence about propofol in GBM. Available laboratory and retrospective clinical studies indicate possible antitumor effects, but the present evidence does not support definitive clinical conclusions. Large prospective and multicenter studies are still needed to clarify the clinical role of propofol in patients with GBM.

## 1. Introduction

Gliomas represent the most common category of primary malignant brain tumors in adults, accounting for approximately 81% of such cases [1–4]. Glioblastoma, also referred to as glioblastoma multiforme (GBM), is a highly aggressive malignant tumor of the central nervous system. It arises from glial cells, such as astrocytes and oligodendrocytes, which provide structural and metabolic support to neurons. GBM is associated with a poor prognosis and limited survival, typically averaging around 15 months following diagnosis [5]. A hallmark of GBM is its elevated oxidative stress, which plays a critical role in tumor progression, therapeutic resistance, and poor response to conventional treatment modalities [6]. Oxidative stress occurs when the generation of reactive oxygen species (ROS) exceeds the capacity of the cellular antioxidant defense system, resulting in damage to critical biomolecules such as DNA, proteins, and lipids [7–10]. The standard treatment protocol for GBM typically begins with surgery, followed by radiotherapy and temozolomide chemotherapy [5, 11, 12]. Currently, surgical intervention remains the primary treatment modality for patients with glioma. Although most patients receive adjuvant radiotherapy and chemotherapy following surgery, high recurrence rates persist, and the long‐term prognosis remains poor [13]. Recent research has increasingly explored the potential impact of anesthetics and analgesics on tumor progression and treatment outcomes [5]. Total intravenous anesthesia (TIVA) has been associated with shorter surgical durations in glioblastoma procedures compared with inhalational anesthesia (IA) [14, 15]. Propofol, a widely used intravenous anesthetic, has been shown to play a key role in various tumor‐related biological processes, exhibiting predominantly antitumor effects alongside occasional cancer‐promoting activity across different malignancies [16]. The use of TIVA with propofol may reduce anesthesia exposure, shorten operative time, and accelerate postoperative recovery in patients undergoing glioblastoma surgery [14].

Furthermore, a retrospective computational study reported that a prognostic model based on propofol anesthesia–related risk scores may have predictive value in patients with glioblastoma. However, external validation and prospective clinical studies are still needed to confirm its clinical relevance and prognostic utility [17, 18].

An increasing number of preclinical studies have examined the effects of propofol on cancer‐related molecular pathways, microRNAs, long noncoding RNAs, and downstream signaling molecules. Some experimental studies have also reported that propofol may increase the response of glioma cells to chemotherapeutic agents and small‐molecule compounds [16, 19]. However, current clinical evidence mainly comes from retrospective studies and perioperative observations, and definitive effects on long‐term oncologic outcomes remain unclear. This review summarizes current preclinical evidence about the molecular effects of propofol in GBM and also discusses available clinical findings related to perioperative and postoperative outcomes.

## 2. Clinical Effects of Propofol

Fang et al. [20] reported that patients in the sevoflurane group (observation group) had significantly shorter anesthesia and extubation times, as well as reduced intraoperative bleeding, compared with the propofol group (control group). No significant differences were observed in surgical duration, total operating room time, fluid intake, or urine output. Postoperative recovery was faster in the sevoflurane group, with earlier eye‐opening and orientation. Cognitive assessments showed higher OAAS scores immediately after extubation and 1 h later, along with significantly better MMSE scores. Additionally, the sevoflurane group experienced fewer postoperative complications, including agitation, urinary tract infections, deep vein thrombosis, and hypertension. Intraoperative electrocorticography (ECoG) is a reliable technique for monitoring cortical electrical activity and identifying epileptogenic foci during glioblastoma‐related epilepsy (GRE) surgery. Li et al. [1] reported that administration of low‐dose propofol significantly enhances ECoG activation in patients with GRE compared with nonepileptic glioma patients. Moreover, propofol‐treated individuals exhibited fewer early and long‐term postoperative seizures, along with a reduced frequency of interictal epileptiform discharges (IEDs). These findings suggest that propofol‐induced ECoG activation may serve as a valuable intraoperative tool for accurately localizing both overt and latent epileptogenic zones. Huang et al. [21] reported that propofol anesthesia was associated with improved survival outcomes and lower postoperative recurrence rates compared with desflurane anesthesia. Dong et al. [22] reported that sevoflurane anesthesia was associated with higher mortality in patients with preoperative Karnofsky Performance Status (KPS) below 80 compared with propofol anesthesia. These findings suggest that propofol may offer clinical advantages for patients with reduced functional status prior to surgery. Cerebral edema is a common postoperative complication following glioma resection. Aquaporin‐4 (AQP4) and aquaporin‐9 (AQP9) play key roles in regulating brain water homeostasis. Yang et al. [23] reported that patients in the propofol group exhibited significantly lower AQP4 expression than those in the sevoflurane group. No significant difference was observed in AQP9 expression between the two groups. These findings suggest that propofol may contribute to reduced cerebral edema by downregulating AQP4 expression (Table 1). However, a recent systematic review and meta‐analysis by Penchev et al. [24] did not report a statistically significant overall survival (OS) or progression‐free survival (PFS) advantage for TIVA compared with IA in all patients with high‐grade glioma, although some subgroup differences were observed across specific cohorts. Therefore, current clinical evidence remains inconclusive, and prospective studies are still needed.

**Table 1:** Clinical effects of propofol on glioblastoma multiforme.

Author/year	Study design	Pertinent findings
Fang (2022) [20]	Clinical retrospective study (*n* = 192)	Sevoflurane was associated with faster recovery and fewer postoperative complications compared with propofol in glioma surgery.
Li (2023) [1]	Clinical retrospective study (*n* = 306)	Propofol‐enhanced ECoG provides a reliable method for epileptic zone identification in glioma‐related epilepsy surgery, improving postoperative seizure control.
Huang (2021) [7]	Clinical retrospective cohort study (*n* = 103)	Patients receiving propofol anesthesia have shown lower rates of postoperative recurrence compared with those undergoing desflurane anesthesia.
Dong (2020) [22]	Clinical retrospective study (*n* = 294)	Propofol may represent a safer anesthetic choice than sevoflurane for glioma patients with reduced preoperative functional capacity (KPS < 80).
Yang (2015) [23]	Clinical retrospective study (*n* = 30)	Propofol demonstrates selective inhibition of AQP4 expression in glioma tissue, suggesting a potential mechanism for its neuroprotective effects in reducing postoperative cerebral edema during glioma resection.

## 3. Propofol Molecular Effects on Glioma

### 3.1. Proliferation, Invasion, Migration, and Signaling Pathways

Experimental evidence from animal models has demonstrated the modulatory effects of propofol on glioma progression, particularly in relation to cellular proliferation, invasion, and angiogenesis. In a rat glioma model, propofol administration influenced tumor morphology, protein expression, and vascular activity. No tumor formation was observed in the sham‐operated group (S), whereas the glioma group (G) exhibited dense accumulation of tumor cells and neovascularization at the tumor margins, along with central necrosis and cavity formation. Most glioma cells in Group G showed positive expression of glial fibrillary acidic protein (GFAP), confirming their astrocytic origin. Similar histopathological features were noted in the propofol‐treated groups (P1 and P2), which received intravenous propofol at doses of 20 and 40 mg/kg/h for 6 h on Day 10 posttumor induction. Although VEGF expression and the number of VEGF‐positive cells were elevated in all tumor‐bearing groups (G, P1, and P2) compared with the sham group, propofol treatment significantly reduced tumor weight and VEGF levels in P1 and P2 relative to Group G, with no significant differences between the two dosage groups. These findings suggest that propofol may attenuate glioma aggressiveness by modulating VEGF‐mediated signaling pathways [25].

In human glioblastoma cell lines (LN229 and U251), propofol was found to suppress proliferation, migration, and invasion while promoting apoptosis. These effects were mediated through downregulation of circMAPK4, which led to increased levels of miR‐622. miR‐622 directly targets HOXA9, a gene associated with glioma progression. Propofol‐induced elevation of miR‐622 resulted in reduced HOXA9 expression, thereby inhibiting tumor aggressiveness. Suppression of miR‐622 reversed the antitumor effects of propofol, confirming the regulatory role of the circMAPK4/miR‐622/HOXA9 axis in glioma cells. This pathway represents a potential molecular mechanism by which propofol exerts its antiglioma activity [26]. Propofol was shown to inhibit proliferation and invasion while promoting apoptosis in U373 glioblastoma cells. These effects were assessed using MTT assay, caspase‐3 activity measurement, and Matrigel invasion assay. Molecular analysis revealed that propofol significantly upregulated microRNA‐218 (miR‐218), which in turn suppressed matrix metalloproteinase‐2 (MMP‐2) expression. Transfection with miR‐218 precursor mimicked the effects of propofol, whereas inhibition of miR‐218 reversed them, confirming its mediating role. These findings suggest that propofol exerts antiglioma effects partly through the miR‐218/MMP‐2 regulatory axis [27]. Propofol inhibits glioma cell migration and invasion by suppressing the PI3K/AKT signaling pathway. In glioma tissues and cell lines, miR‐206 expression is downregulated; however, propofol treatment significantly upregulated miR‐206 levels. Overexpression of miR‐206 enhanced the inhibitory effect of propofol on PI3K/AKT pathway activation. ROCK1 was identified as a direct target of miR‐206, with its expression elevated in glioma cells but reduced following propofol exposure. Rescue experiments confirmed that the miR‐206/ROCK1 axis mediates the antimigratory and anti‐invasive effects of propofol via PI3K/AKT pathway inhibition [28]. Propofol suppresses glioma cell growth and metastasis by modulating the circNCAPG/miR‐200a‐3p/RAB5A axis. In glioma tissues and cell lines, circNCAPG and RAB5A were significantly upregulated, whereas miR‐200a‐3p expression was downregulated. Treatment with propofol reversed this pattern, increasing miR‐200a‐3p levels and reducing circNCAPG and RAB5A expression. Functional assays demonstrated that overexpression of circNCAPG counteracted the antiproliferative, anti‐invasive, and proapoptotic effects of propofol in vitro. Mechanistically, circNCAPG acts as a competing endogenous RNA (ceRNA), sponging miR‐200a‐3p and thereby promoting RAB5A expression. In vivo experiments further confirmed that propofol inhibits glioma tumor growth by targeting this regulatory pathway [29]. In glioma cell lines U251 and A172, the 24‐h treatment with increasing concentrations of propofol resulted in a dose‐dependent inhibition of cell proliferation, migration, and invasion. Molecular analysis revealed that propofol upregulated miR‐410‐3p expression while downregulating transforming growth factor‐*β* receptor type 2 (TGFBR2). TGFBR2 was identified as a direct target of miR‐410‐3p, and functional assays demonstrated that suppression of miR‐410‐3p reversed the antitumor effects of propofol. However, knockdown of TGFBR2 restored these inhibitory effects, confirming the regulatory role of the miR‐410‐3p/TGFBR2 axis. These findings suggest that propofol exerts its antiglioma activity, at least in part, by modulating this molecular pathway [30]. Laboratory analysis of glioma tissue samples revealed that propofol inhibits glioma cell survival by inducing apoptosis and suppressing proliferation. It also significantly reduced cell migration and invasion. SETBP2‐AS1 expression was elevated in glioma tissues but decreased following propofol treatment. Overexpression of SETBP2‐AS1 attenuated the antitumor effects of propofol, suggesting its involvement in glioma progression. Mechanistically, SETBP2‐AS1 acts as a molecular sponge for microRNA‐373‐3p, which in turn targets pyruvate dehydrogenase kinase 3 (PDK3). Upregulation of miR‐373‐3p reversed the oncogenic effects of SETBP2‐AS1, whereas its inhibition restored PDK3 expression and negated propofol′s suppressive impact on glioma growth. These findings indicate that propofol impedes glioma progression through the SETBP2‐AS1/miR‐373‐3p/PDK3 regulatory axis [31]. Dose‐dependent treatment with propofol significantly inhibited migration and invasion of U251 glioma cells [32]. Molecular analysis revealed that propofol upregulated miR‐622 expression, which in turn suppressed the protein levels of activating transcription factor 2 (ATF2). Transfection with miR‐622 inhibitor reversed this effect, confirming that miR‐622 negatively regulates ATF2. These findings suggest that propofol exerts its antimigratory and anti‐invasive effects in glioma cells through the miR‐622/ATF2 axis. This molecular insight may inform anesthetic selection strategies in glioma‐related surgical procedures [33]. Propofol treatment significantly inhibited proliferation, migration, and invasion of U251 glioma cells, and induced cell cycle arrest and apoptosis. These effects were accompanied by upregulation of the long noncoding RNA GAS5. Functional analysis indicated that downregulation of GAS5 attenuated the antitumor effects of propofol. Mechanistically, propofol promoted GAS5 expression, which in turn increased P21 levels and suppressed the expression of oncogenic markers c‐Myc and GSTM3 [34]. Propofol significantly inhibited the viability, migration, and invasion of C6 glioma cells, accompanied by a reduction in glutamate release. These antitumor effects were reversed by N‐acetylcysteine (NAC), an activator of the cystine/glutamate antiporter system xc^−^. Notably, propofol continued to suppress cell proliferation even in the presence of high extracellular glutamate (100 *μ*M). At the molecular level, propofol upregulated membrane expression of GluR2 and downregulated xCT, changes that were reversed by (R,S)‐AMPA, a Ca^2+^‐permeable AMPA receptor agonist. These findings suggest that propofol exerts its antiglioma effects primarily through modulation of the CPAR–system xc^−^ signaling axis [35]. In a rat model of glioma using C6 cells, propofol treatment significantly reduced cell viability, migration, and invasion compared with controls. Molecular analysis showed that propofol upregulated nuclear ADAR2 expression and increased membrane localization of the AMPA receptor subunit GluR2. These effects were maintained in cells transfected with negative control siRNA. However, silencing ADAR2 via siRNA reversed the antitumor effects of propofol, leading to increased cell viability, migration, and invasion, along with decreased ADAR2 and GluR2 expression. These findings suggest that propofol suppresses glioma cell aggressiveness through activation of the ADAR2/GluR2 signaling pathway [36].

Xu et al. [37] reported that propofol may inhibit the Wnt/*β*‐catenin signaling pathway in glioma models. In 293T cells transfected with Wnt1 or Wnt3 plasmids, or exposed to Wnt3A‐conditioned medium, propofol reduced SuperTopFlash reporter activity and lowered the expression of Wnt target genes. In glioma cells, propofol also reduced pLRP6 and total *β*‐catenin protein levels. In vitro and xenograft experiments showed lower glioma cell proliferation after propofol treatment, while no significant changes were observed in mouse body weight. Xenograft models also showed lower Wnt activity after propofol exposure. These findings may provide a mechanistic basis for further study of propofol in glioma biology.

Li et al. [38] reported that propofol may suppress glioma progression through the circ_0047688/miR‐516b‐5p/IFI30 axis. Propofol reduced circ_0047688 expression in a dose‐dependent manner and decreased glioma cell proliferation. Silencing circ_0047688 increased the inhibitory effects of propofol, whereas circ_0047688 overexpression reduced these effects in vitro and in vivo. circ_0047688 acted as a molecular sponge for miR‐516b‐5p, which targeted IFI30, a gene related to glioma aggressiveness. Propofol treatment restored miR‐516b‐5p activity and reduced IFI30 expression. These changes were associated with lower glioma cell growth, migration, and invasion. These findings may provide a mechanistic basis for future studies on propofol‐related molecular pathways in glioma. GABAA receptors are pentameric ion channels that allow chloride influx after GABA activation. The U3047MG cell line has been used to study GABA‐A receptor pharmacology in glioblastoma‐derived cells. Babateen et al. [39] reported that whole‐cell current analysis showed a half‐maximal effective concentration (EC50) of 36 *μ*M for GABA‐activated currents. THIP activated these currents, whereas diazepam (1 *μ*M), etomidate, and propofol (50 *μ*M) increased their activity. In addition, 1 *μ*M diazepam, 50 *μ*M propofol, and 100 nM etomidate increased the effect of 10 *μ*M GABA to different degrees. Overall, these preclinical findings suggest that high micromolar concentrations of propofol may interact with GABAA receptors, whereas diazepam mainly affects these receptors in the presence of GABA [40–42] (Table 2).

**Table 2:** Molecular effects of propofol on glioma.

Target/pathway	Effect of propofol	Experimental model	Key findings	Author/year
circMAPK4/miR‐622/HOXA9 axis	↓circMAPK4 → ↑miR‐622 → ↓HOXA9 → antitumor	U251, LN229 cells (5 *μ*g/mL, 24 h)	Propofol inhibits proliferation, migration, invasion; induces apoptosis via this axis. Confirmed by luciferase/RIP assays.	Xiao (2023) [26]
miR‐218/MMP‐2 axis	↑miR‐218 → ↓MMP‐2 → anti‐invasion, proapoptosis	U373 cells (MTT, Matrigel assays)	Propofol inhibits proliferation/invasion and induces apoptosis via miR‐218. Anti–miR‐218 reverses these effects.	Xu (2015) [27]
miR‐206/ROCK1/PI3K‐AKT axis	↑miR‐206 → ↓ROCK1 → ↓PI3K/AKT → antimetastasis	Glioma cells (MTT, Transwell assays)	Propofol inhibits migration/invasion via miR‐206 targeting ROCK1, suppressing PI3K/AKT. Rescue assays confirm axis.	Wang (2020) [28]
circNCAPG/miR‐200a‐3p/RAB5A axis	↓circNCAPG → ↑ miR‐200a‐3p → ↓RAB5A → antimetastasis	Glioma cells (CCK‐8, Transwell), xenografts	Propofol inhibits growth/metastasis via circNCAPG sponging miR‐200a‐3p to downregulate RAB5A. Confirmed by dual‐luciferase/RIP.	Zhang (2021) [29]
miR‐410‐3p/TGFBR2 axis	↑miR‐410‐3p → ↓TGFBR2 → antimetastasis	U251, A172 cells (MTT, Transwell)	Propofol inhibits growth/metastasis via miR‐410‐3p targeting TGFBR2. Rescue experiments confirm axis.	Li (2020) [32]
SBF2‐AS1/miR‐373‐3p/PDK3 axis	↓SBF2‐AS1 → ↑miR‐373‐3p → ↓PDK3 → antitumor	21 glioma tissues + cell assays (MTT, Transwell)	Propofol inhibits viability/metastasis via lncRNA/miR metabolic axis. Rescue experiments confirm PDK3 role.	Jiang (2023) [31]
miR‐622/ATF2 axis	↑miR‐622 → ↓ATF2 → antimetastasis	U251 cells (1‐20 *μ*g/mL, 6 h)	Propofol inhibits invasion/migration dose‐dependently via miR‐622 targeting ATF2. miR‐622i reverses effects.	Wang (2018) [33]
GAS5/P21/c‐Myc/GSTM3 axis	↑GAS5 → ↑P21 and ↓cMyc/GSTM3 → cell cycle arrest	U251 cells (siRNA knockdown)	Propofol upregulates tumor‐suppressive lncRNA GAS5, activating P21 while inhibiting oncogenic c‐Myc/GSTM3. Effects reversed by GAS5 siRNA.	Cheng (2022) [34]
CPAR‐system xc − pathway	↑ GluR2 and ↓ xCT → ↓ Glutamate release → antitumor	C6 cells (NAC/AMPA rescue)	Blocks glutamate‐driven invasion via AMPA receptor modulation and xCT suppression.	Wang (2017) [35]
ADAR2‐GluR2 axis	↑ ADAR2 → ↑ GluR2 → anti‐invasion	C6 cells (ADAR2‐siRNA rescue, 1.2 *μ*g/mL, 6 h) (MTT, Transwell) rats	Propofol upregulates RNA‐editing enzyme ADAR2 to enhance GluR2 surface expression, inhibiting metastasis. siRNA knockdown reverses effects.	Wang (2016) [36]
Wnt/*β*‐catenin signaling	↓Wnt1/Wnt3A → ↓ *β*‐catenin → antiproliferation	293 T cells + glioma cells + xenografts (mice)	Propofol significantly inhibited the transcriptional activity of the SuperTopFlash reporter and the expression of Wnt target genes.	Xu (2018) [37]
circ_0047688/miR‐516b‐5p/IFI30 axis	↓ circ_0047688 → ↑ miR‐516b‐5p → ↓ IFI30 → antitumor	Glioma cells + xenografts (CCK‐8, EdU, Transwell) mouse xenograft model	Propofol disrupts circRNA sponging of miR‐516b‐5p, suppressing IFI30‐mediated oncogenesis. Overexpression reverses effects.	Li (2023) [1]
GABAA receptor activation	Positive allosteric modulation (*α*3*β*3*θ* subunits) → Cl^−^ influx	U3047MG cells (patch‐clamp, EC_50_ = 50 *μ*M)	Enhances GABAergic currents (EC_50_ = 36 *μ*M GABA). Synergizes with etomidate (EC_50_ = 55 nM).	Babateen (2015) [39]

Abbreviations: circMAPK4, circular RNA mitogen‐activated protein kinase 4 (sponges miR‐622); GAS5, growth arrest‐specific 5 lncRNA, a tumor suppressor in gliomas; GSTM3, glutathione S‐transferase mu 3, associated with temozolomide resistance; HOXA9, homeobox A9, an oncogenic transcription factor in glioma; MMP‐2, matrix metalloproteinase‐2, critical for glioma invasion; PDK3, pyruvate dehydrogenase kinase 3 (glycolytic regulator); ROCK1, Rho‐associated kinase 1, promotes cytoskeletal remodeling in invasion; SBF2‐AS1, lncRNA SET binding factor 2‐antisense strand 1.

Fan et al. [43] reported that propofol may increase stem‐like features in glioma models through GABAA receptor pathways. Propofol exposure caused higher Src expression and higher ZDHHC5 palmitoylation, followed by higher EZH2 and Oct4 levels. These molecular changes were linked with stem‐like properties in glioma cells through the GABAA–Src–ZDHHC5–EZH2 pathway. These findings raise a possible safety concern in preclinical glioma models, but current evidence does not prove a direct link with tumor recurrence or disease progression in patients with GBM. Edgunlu et al. [44] reported that propofol induced apoptosis in U‐87 MG glioma cells compared with untreated control cells. Propofol altered the expression of several genes in the PI3K/AKT pathway, and these molecular changes may be linked with apoptosis in U‐87 MG cells. Li et al. [45] reported that propofol lowered proliferation, migration, and invasion in U87 glioma cells after 48 h of treatment. Lower MMP‐2, MMP‐9, Ki‐67, p‐Akt/Akt, and p‐PI3K/PI3K levels were observed after propofol exposure, whereas miR‐134 expression showed higher levels. Higher propofol concentrations (2.00–5.00 mmol/L) produced stronger inhibitory effects than lower concentrations. The 5 mmol/L group showed lower proliferation, migration, and invasion than the 2 mmol/L group. Lower MMP‐2, MMP‐9, Ki‐67, p‐Akt/Akt, and p‐PI3K/PI3K levels were also observed in this group, whereas miR‐134 expression showed higher levels. These findings suggest that propofol may lower glioma cell aggressiveness through miR‐134 upregulation and partial inhibition of the PI3K/Akt pathway in preclinical models.

### 3.2. Antioxidant Effect

Elevated oxidative stress promotes invasive and metastatic potential in cerebral neoplasms. Divalent metal transporter 1 (DMT1) activation, which is modulated by glutamatergic receptor signaling, contributes to enhanced oxidative stress conditions that may facilitate oncogenesis and tumor progression. To investigate the molecular mechanism underlying propofol‐mediated oxidative stress reduction in glioma models, C6 glioma‐bearing male Wistar rats (induced by intracranial injection of C6 cells) received propofol treatment or served as untreated controls for 6 h before sacrifice. The proposed mechanistic hypothesis suggests that propofol may alleviate oxidative stress in gliomas by inhibiting the Ca^2+^‐permeable AMPA receptor (CPAR)‐DMT1 signaling axis. Studies revealed that propofol infusion (20–40 mg/kg/h) significantly upregulated GluR2 expression while downregulating DMT1 and depleting glutathione levels in peripheral tumor regions compared with the glioma core. In vitro experiments demonstrated that (R, S)‐AMPA treatment enhanced both ROS creation and the expression of DMT1, effects that were partially attenuated by propofol coadministration. These results indicate that propofol exerts its antitumor effects through CPAR‐mediated regulation of DMT1, ultimately reducing oxidative stress and inhibiting glioma cell proliferation [46, 47].

Propofol at levels ranging from 400 to 1000 *μ*M increased the influx of calcium ions into DBTRG‐05MG cells in a manner that depended on the concentration. The increase in calcium ions prompted by propofol remained unchanged despite the presence of nifedipine, econazole, SK&F96365, and protein kinase C (PKC) activators, but was reduced when a PKC inhibitor was used. With concentrations between 300 and 700 *μ*M, propofol caused cell death in a way that depended on the dosage. Additionally, Annexin V/PI staining showed that propofol concentrations of 300–500 *μ*M triggered programmed cell death or apoptosis. Propofol also significantly raised the production of ROS. In summary, propofol led to a rise in calcium ion levels by initiating calcium release from the endoplasmic reticulum in ways dependent on PLC and PKC, while also encouraging calcium entry through channels not reliant on store operation. The cell death associated with propofol might be related to apoptosis driven by ROS formation [48] (Figure 1).

**Figure 1:**
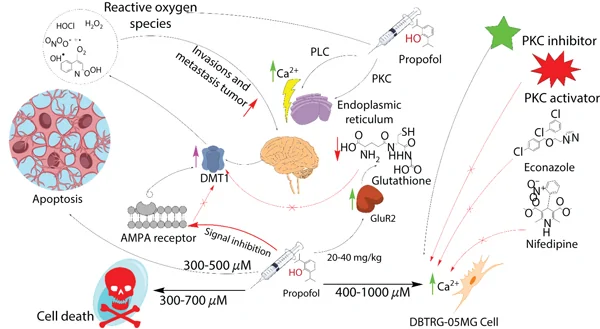
Antioxidant effect of propofol on GBM. Propofol induces calcium influx in DBTRG‐05MG glioblastoma cells via PLC‐ and PKC‐dependent pathways, triggering ROS generation and dose‐dependent apoptosis. The process involves endoplasmic reticulum calcium release, nonstore‐operated calcium entry, and modulation of the CPAR–DMT1 axis. Propofol also alters GluR2 and glutathione levels, contributing to oxidative stress regulation and glioma cell death.

### 3.3. Impact on Immune Checkpoints and Molecular Subtypes of Glioma

Cen et al. [49] compared propofol and sevoflurane anesthesia in relation to molecular subtype markers and immune‐checkpoint expression in GBM. The study reported that propofol anesthesia was linked with lower SERPINI1 and CAMK2A expression compared with sevoflurane anesthesia. SERPINI1 and CAMK2A were also related to clinical outcomes in patients with GBM and lower grade glioma, and these proteins may have tumor‐suppressive roles. In addition, propofol anesthesia was associated with higher PD‐L1 expression in glioma tissue. The clinical significance of this finding remains unclear because PD‐L1 expression in GBM may have complex effects on tumor immunity and immune‐checkpoint responses. Recent studies suggest that PD‐L1 signaling in GBM may have heterogeneous effects on immune escape, macrophage activity, and response to immune‐checkpoint therapy, which complicates interpretation of PD‐L1 changes after anesthetic exposure [50].

### 3.4. Effect on the Sensitivity of Glioblastoma Cells to Temozolomide

Findings indicate that propofol increased the response of temozolomide‐resistant glioblastoma xenograft and cell models to temozolomide treatment. In TMZ‐resistant GBM xenografts, higher HIF‐1*α*, MGMT, p‐p65, and Cox2 levels were observed compared with standard GBM xenografts. Combined treatment with propofol lowered the expression of these proteins. In cell experiments, conditioned medium from M0 macrophages increased U251 cell survival, inflammatory responses, and HIF‐1*α*, MGMT, p65, and Cox2 expression, whereas apoptosis showed lower levels. Propofol reduced PMA‐induced THP‐1M0 macrophage activation, and conditioned medium from propofol‐treated macrophages reversed the effects of untreated M0 macrophage medium on U251 cells. DMOG treatment also reversed the effects caused by propofol‐treated macrophage medium on U251 cells [51]. These findings come from TMZ‐resistant xenograft and in vitro glioma models. Current evidence does not prove similar effects in clinical GBM outcomes or patient survival.

Cheng et al. [52] reported that propofol may increase the sensitivity of U251 glioma cells to temozolomide through the lncRNA GAS5 pathway. Combined treatment with propofol and temozolomide lowered cell survival and invasion, whereas apoptosis showed higher levels. Higher GAS5 and P21 expression and lower c‐Myc and GSTM3 levels were also observed after combined treatment. GAS5 silencing partially reversed these effects, which suggests that GAS5 may have a role in the response of glioma cells to propofol and temozolomide treatment in preclinical models.

## 4. Discussion

Current evidence suggests biological plausibility, but clinical support remains limited and inconsistent. A recent systematic review and meta‐analysis found no clear pooled OS or PFS benefit for TIVA compared with IA, although survival findings differed across anesthetic comparators and glioma cohorts [24]. Some retrospective cohorts reported perioperative advantages with propofol‐based anesthesia, such as shorter operative time or improved recovery profiles [14], but Fang et al. [20] reported shorter anesthesia and extubation times, faster early recovery, better early consciousness/cognitive scores, and fewer selected postoperative complications with sevoflurane compared with propofol. Propofol has also been associated with ECoG activation and fewer postoperative seizures in one retrospective study, whereas OS and PFS findings remain inconsistent across different cohorts [1, 21, 53]. This seizure‐related finding should be read as a perioperative benefit, not as proof of tumor control or survival improvement. In addition, studies used different anesthetic comparators, mainly sevoflurane or desflurane, which may contribute to heterogeneity in reported outcomes. Most available studies also have limitations such as single‐center design, small sample size, retrospective analysis, and variable follow‐up periods. Therefore, prospective multicenter studies are still needed. Mughal et al. [54] also noted that current evidence mainly comes from retrospective cohorts with heterogeneous anesthetic protocols and limited control of molecular and clinical confounders.

Future studies should examine patients according to age, KPS, extent of resection, MGMT methylation, IDH status, and radiogenomic characteristics because GBM shows marked molecular heterogeneity [55, 56]. Precise recording of propofol infusion rates and plasma concentrations is also necessary because many in vitro studies used prolonged micromolar exposure conditions that may not fully reflect intraoperative exposure. Additional pharmacokinetic studies with blood and tumor tissue sampling during glioma surgery may help clarify intratumoral drug exposure and dose‐related biological effects [38, 47].

Laboratory studies point to several antitumor mechanisms: Propofol can reduce PI3K/AKT and Wnt signaling, increase tumor‐suppressive miRNAs such as miR‐206, miR‐410‐3p, and miR‐622, lower oxidative stress through the CP–AMPA–DMT1 axis, and restore temozolomide sensitivity in resistant models by reducing HIF‐1*α* and MGMT [32, 37, 46, 51]. These pathways rarely act in isolation, so the next frontier lies in multiomic experiments—transcriptome, proteome, and metabolome—performed on patient‐derived organoids or orthotopic xenografts. With those platforms, researchers can test propofol in short intraoperative pulses, prolonged postoperative sedations, or repeated exposures that mimic staged surgeries, then ask which schedule locks in durable epigenetic or transcriptomic shifts. Such models can also test propofol with radiotherapy, PARP inhibitors, IDH inhibitors, or immune checkpoint blockers, especially after reports that propofol upregulates PD‐L1 expression and reshapes macrophage activation [49] (Figure 2).

**Figure 2:**
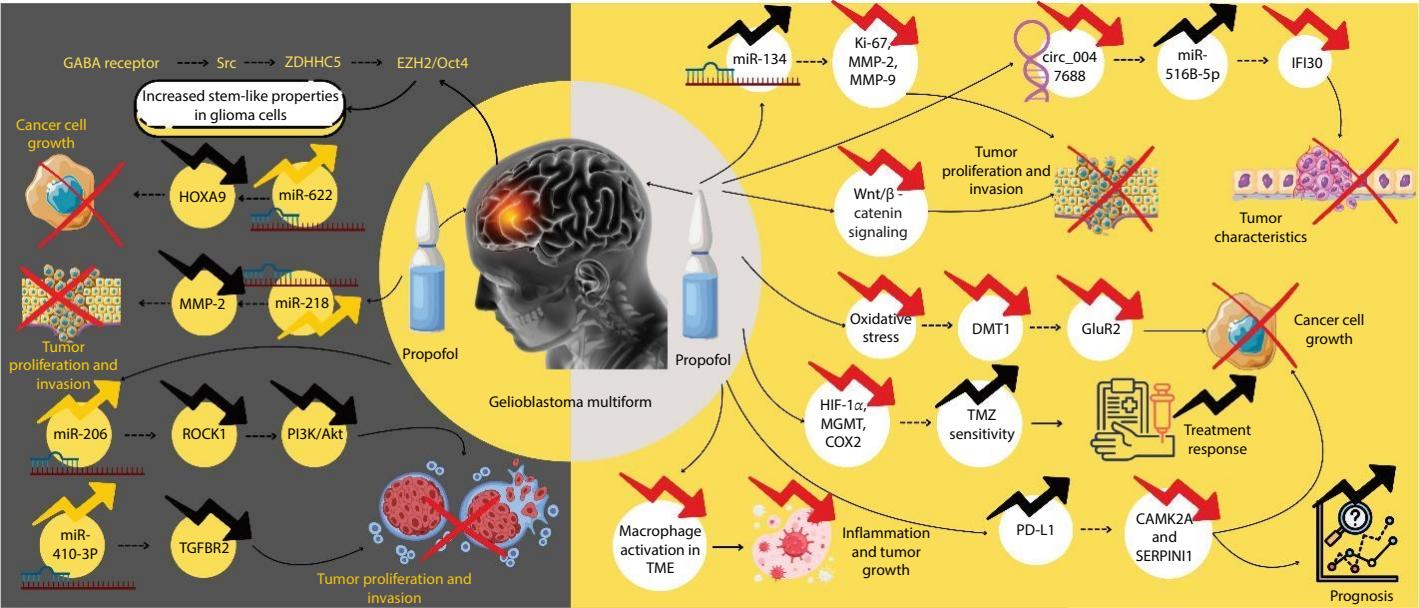
Mechanisms and molecular factors types affected by propofol in GBM. Schematic representation of propofol′s molecular effects on glioblastoma multiforme (GBM). Propofol downregulates oncogenic pathways (e.g., HOXA9, ROCK1, and PI3K/AKT) and upregulates tumor‐suppressive signals (e.g., miR‐134 and miR‐218), modulating cell proliferation, invasion, inflammation, and treatment response. The GABA receptor–Src–ZDHHC5–EZH2 axis may contribute to stem‐like properties in glioma cells.

Propofol may increase Src activity, ZDHHC5 palmitoylation, and EZH2/Oct4 expression in experimental glioma models. This finding is a true counter‐signal because stem‐like features are closely related to recurrence and therapy resistance in GBM biology [43]. Defining the exposure window that flips this switch, and learning whether coadministration of Src or EZH2 inhibitors prevents it, represents critical safety tasks. Animal work should also examine neurocognitive outcome because GBM patients already face radiotherapy‐induced decline, and any anesthetic strategy must avoid extra harm. Digital infrastructure can speed discovery. Large anesthesia‐information systems link dose, depth‐of‐anesthesia indices, hemodynamics, and recovery markers; when merged with institutional cancer registries and national survival datasets, they can reveal subtle dose–response curves and long‐term patterns that trials may miss. Biobanks that freeze perioperative serum, cerebrospinal fluid, and resected tumor will let investigators connect intraoperative drug exposure with later molecular drift, clonal evolution, and immune micromilieu. Finally, machine‐learning tools that integrate imaging radiomics with anesthetic variables may predict who benefits most from propofol and thus steer personalized anesthesia plans. In short, propofol now sits at an interesting intersection of neuroanesthesia and neuro‐oncology: the clinical evidence suggests possible perioperative value, the laboratory data support biological activity, and only coordinated trials, better matched bench models, and transparent data sharing can clarify whether this everyday anesthetic has any disease‐modifying role in GBM care.

## 5. Conclusion

Propofol may have perioperative value in glioma surgery, particularly for seizure‐related monitoring, ECoG activation, and selected recovery or safety outcomes reported in retrospective cohorts. However, these clinical findings should not be read as proof of better tumor control. Current evidence does not confirm a clear OS or PFS benefit for propofol‐based TIVA compared with IA in high‐grade glioma surgery. The laboratory data support biological activity through PI3K/AKT, Wnt, miRNA‐related pathways, oxidative stress regulation, and TMZ sensitivity, but these effects remain mainly preclinical. At the same time, the GABAAR‐Src‐ZDHHC5‐EZH2 pathway and PD‐L1‐related findings show that propofol biology in GBM is not uniformly favorable. Overall, propofol should be viewed as a biologically active anesthetic with possible perioperative benefits, whereas its disease‐modifying role in GBM remains unproven.

## 6. Limitations and Recommendations

The clinical studies have several weaknesses. Most studies were single center and included relatively small patient groups. Other studies had retrospective design, and follow‐up periods were not consistent across reports. The anesthetic agents also differed between studies, with comparisons made against either sevoflurane or desflurane. These differences make direct comparison difficult. Large prospective studies with the same comparator and clearly defined clinical outcomes would provide more reliable evidence. Factors such as age, KPS, tumor characteristics, extent of resection, MGMT methylation, and IDH status were not handled in the same way across published cohorts. Some reports adjusted for these variables, whereas others did not. Future research should classify patients according to these features and report adjusted outcome analyses in a consistent format. Several cell culture studies exposed glioma cells to propofol concentrations that may not reflect the levels reached during routine neurosurgical anesthesia. Blood and tumor samples collected during surgery could help connect tissue drug concentrations with the molecular changes observed after exposure. Patient‐derived organoids, orthotopic glioma models, and multiomic analyses could provide a more realistic picture of how propofol acts under conditions that resemble clinical practice. Some molecular findings also need careful confirmation. More studies are needed to confirm these observations and to determine whether these molecular changes influence treatment response or long‐term outcome.

## Funding

No funding was received for this manuscript.

## Disclosure

The authors take full responsibility for the accuracy, integrity, and final content of the manuscript.

## Conflicts of Interest

The authors declare no conflicts of interest.

## Data Availability

No datasets were generated or analyzed during the current study.
